# Novel homozygous nonsense mutation in glucagon‐like peptide‐2 receptor gene resulting in severe human illness

**DOI:** 10.1002/jpr3.12125

**Published:** 2024-09-05

**Authors:** Claire Jaramishian, Shivani Kamal, Martín G. Martín, Nathan Zev Minkoff

**Affiliations:** ^1^ Pediatric Residency Program Valley Children's Healthcare Madera California USA; ^2^ Department of Pediatrics, Division of Gastroenterology and Nutrition, Mattel Children's Hospital and the David Geffen School of Medicine University of California Los Angeles Los Angeles California USA; ^3^ Department of Pediatric Gastroenterology, Hepatology & Nutrition Valley Children's Healthcare Madera California USA

**Keywords:** diarrhea, genetic mutation, genetic/metabolic gi disease, intestinal failure, pediatric

## Abstract

Glucagon‐like peptide‐2 (GLP2) acts on the GLP2 receptor (GLP2R) and plays a role in intestinal growth and adaptation. The endogenous actions of GLP2R do not have an established association with human disease, although mouse‐knockout models in a stressed state show enhanced susceptibility to small bowel injury, increased morbidity, mortality, and abnormal host–bacterial interactions. We report an 11‐month‐old female with multiple intensive care unit admissions for severe metabolic acidosis due to profuse nonbloody diarrhea in the context of various infections. She had normal growth, lab testing, and stooling patterns between illnesses. Trio‐whole genome sequencing revealed homozygous nonsense variants resulting in nonfunctional GLP2R. This is the first known human documented with a GLP2R‐deficient phenotype, resulting in clinical illness, which correlates with the findings in the GLP2R mouse knockout model and furthers our understanding of GLP2R and the action of teduglutide, a GLP2 analog used for the treatment of short bowel syndrome.

## 1 INTRODUCTION

The glucagon‐like peptide‐2 receptor (GLP2R) is a G‐protein‐coupled receptor involved in intestinal growth and adaptation, which has gained increasing attention with the use of teduglutide, a recombinant glucagon‐like peptide‐2 (GLP2) analog, for children and adults with short bowel syndrome.^1^, ^2^

In murine models, expression of Glp2r has been localized to the intestinal tract, cerebral cortex, mesenteric lymph nodes, gallbladder, urinary bladder, mesenteric fat, and testes.^3^ GLP2R function has not been established in humans, as a monogenic biallelic loss‐of‐function variant has yet to be described. A generalized *Glp2r* mouse‐knockout model exhibited normal growth at baseline.^4^ However, compared to control mice, *Glp2r*‐null mice were more susceptible to small bowel epithelial injury following either administration of indomethacin or irinotecan, resulting in increased morbidity and mortality, and abnormal host–bacterial interactions, including bacterial overgrowth.^4^ These findings suggest gastrointestinal differences in mice during homeostasis were not discernible between *Glp2r*‐null and control mice; however, induced small bowel epithelial injury had a more pronounced effect in *Glp2r*‐null mice.

## 2 CASE REPORT

We report the case of a former term 11‐month‐old female of Mixtecan background with a history of two pediatric intensive care unit admissions and four floor admissions for severe metabolic acidosis and hypernatremic dehydration, with hyperchloremia secondary to recurrent acute nonbloody diarrhea in the context of various viral and bacterial infections. These infections include cytomegalovirus, norovirus, severe acute respiratory syndrome coronavirus 2 (coronavirus disease 2019), and Enteropathogenic *Escherichia coli*. The duration of her viral infections were typical. However, they were all associated with severe diarrhea.

The proband had a normal prenatal course with no complications during delivery and a documented birth weight of 2.267 kg, the fifth percentile weight for age. Family history was noncontributory and her parents denied the possibility of consanguinity; they were from the same small town in Guerrero, Mexico, where isolated founder variants might predispose to an autosomal recessive disorder.

For all admissions, the patient presented hypotensive, tachycardic, and tachypneic, both with and without fever. The physical exam was notable for a flat fontanel, dry mucous membranes, and Kussmaul respirations. She demonstrated good interval growth between her admissions, with a weight for age *z* score improving from −3.21 to −1.59. Her serum laboratory testing showed a sodium of 177 mmol/L (ref: 134–145 mmol/L), chloride >150 mmol/L (ref: 98–110 mmol/L), and bicarbonate of 9 mmol/L (ref: 21–32 mmol/L). Extensive clinical evaluations were obtained by various subspecialties including genetics, gastroenterology, and immunology. Normal results included a newborn screen, metabolic testing (including urine organic acids), a congenital diarrhea genetic panel, assessments for autoimmune enteropathies, and immunodeficiency‐related testing, including human immunodeficiency virus, immunoglobulins, and a lymphocyte subset panel. While feeding, her stool electrolytes were consistent with osmotic diarrhea (sodium 31 mmol/L, potassium 24 mmol/L, and chloride <20 mmol/L).^5^ An upper endoscopy was visually normal with microscopic findings of villous blunting with increased intraepithelial lymphocytes and lamina propria cellularity of the duodenum. Sigmoidoscopy was grossly and histologically normal.

On her first admission, she was 7 weeks old and received 7 days of parenteral nutrition in the setting of growth faltering and severe diarrhea. During each subsequent admission, she received a combination of intravenous fluids containing saline, acetate, dextrose, and oral nutrition, with close monitoring of her electrolytes and stool output. At all admissions, she continued having diarrhea while nil per os, before the volume of stool improved before discharge.

She was clinically well between admissions. During these periods of wellness, she had appropriate weight gain, a normal stooling pattern, and normal serum electrolytes. A geneticist was consulted and recommended trio genome sequencing, which revealed a homozygous nonsense variant in the *GLP2R* (c.1249 C > T, p.R417Ter; rs543002333). The *GLP2R* variant results in a truncated protein with nonsense‐mediated decay that is likely nonfunctional. The rare variant has a heterozygote allelic frequency (0.00001807) and is not observed in the homozygous state in gnomAD version 4.0. The patient's mother was also heterozygous for the same variant (p.R417Ter) of the *GLP2R* gene. No other significant genetic anomalies were noted and genetic counseling was provided to the family.

We recommended that the patient wear a medical alert bracelet and that her parents carry a medical alert note explaining the patient's diagnosis to give to any healthcare provider caring for her. We also requested that if she develops diarrhea, she has intravenous fluids started immediately, and preparations be made for admission, potentially to the intensive care unit, to minimize the severity of her decompensation.

At the time we were last in contact with the family, our patient was 17 months old, had not had any hospital admissions for 7 months, and had adequate growth with a weight for age *z* score of −0.39. No further comorbidities had been identified. It is unclear why, after a time of repeated admissions, she had been subsequently healthy and without severe diarrhea or metabolic acidosis.

## 3 DISCUSSION

There have been no previously published reports of monogenic homozygosity for a nonsense or missense mutation in the *GLP2R* gene. Therefore, this is the first known human documented with a *GLP2R*‐deficient phenotype resulting in clinical illness to our knowledge. We suspect this patient's lack of functioning GLP2R may have contributed to the phenotype of normal growth and normal stooling habits when well but severe/life‐threatening diarrhea, resulting in dehydration and hypernatremic hyperchloremic metabolic acidosis during periods of illnesses. This further postulates the relationship between the microbiota and Glp2, as prebiotic and probiotic administration in mouse models altered the expression of Glp2, thereby altering gut permeability and production of pro‐inflammatory cytokines.^6^ The GLP2R is the target of the medication teduglutide, a GLP2 analog used in pediatric and adult patients with intestinal failure secondary to short bowel syndrome,^2^ yet much remains unknown about the in vivo function of this receptor outside of mouse models. This case furthers our understanding of this receptor's endogenous in vivo function by demonstrating the disease pattern that results from its absence in a young child. We believe that additional functional and microscopic analysis of human intestines could contribute to a better understanding of GLP2R biology.

## CONFLICT OF INTEREST STATEMENT

The authors declare no conflict of interest.

## ETHICS STATEMENT

Informed patient consent was obtained from the patient's family for publication of the case details.
